# Sequential Pyruvic Acid Pretreatment and Alkaline Extraction of Bamboo Leaf Phenolics: Two-Stage Mass Transfer Kinetics

**DOI:** 10.3390/foods15152655

**Published:** 2026-07-28

**Authors:** Yongkang Mo, Xiaoying Liu, Jingpeng Zhou, Jianquan Ren, Baojie Liu, Chengrong Qin, Chen Liang, Shuangquan Yao

**Affiliations:** 1 Guangxi Key Laboratory of Clean Pulp & Papermaking and Pollution Control, School of Light Industrial and Food Engineering, Guangxi University, Nanning 530004, China; 2 Shandong Huatai Paper Co., Ltd., Dongying & Shandong Key Laboratory of Biobased Material and Green Pulp Papermaking, Dongying 257335, China

**Keywords:** bamboo leaves, total phenolics, pyruvic acid pretreatment, alkaline extraction, mass transfer kinetics, intraparticle diffusion

## Abstract

This study examined total phenolic release and mass transfer during sequential pyruvic acid (PA) pretreatment and sodium hydroxide (NaOH) extraction of bamboo leaves. A 4 × 4 design comprised PA concentrations of 7.0, 8.0, 9.0, and 10.0% and NaOH concentrations of 3.0, 4.0, 5.0, and 6.0%. Kinetic curves from 40 to 120 min showed rapid release followed by a gradual approach to equilibrium. Normalized conversion F(t) was used to evaluate release progression, and two consistent fitting windows were applied for parameter estimation. The LDF model described the 40, 60, and 80 min data and provided *k*_f_, while the Crank spherical diffusion model described the 80, 100, and 120 min data and provided *D*_eff_. These overlapping windows represent different portions of the same continuous extraction process rather than physically separate stages. Model comparison and predictive validation supported the framework. The characteristic time ratio *τ*_m_/*τ*_d_ exceeded 2 under all tested conditions, indicating that external liquid film transfer contributed the greater relative resistance. The condition of 8.0% PA and 5.0% NaOH provided the most balanced performance, yielding 18.55 ± 0.26 mg GAE/g, equivalent to 94.4% of the maximum. These results provide a practical basis for rate control analysis and condition selection.

## 1. Introduction

Polyphenols, a major class of plant-derived secondary metabolites, have attracted considerable attention because of their diverse bioactivities and health-promoting potential. They have been widely explored for food-related applications, particularly as natural antioxidants and food preservation agents [1,2,3]. As an important reservoir of natural bioactive compounds, polyphenols are widely distributed in plant tissues such as fruits, leaves, seed coats, and bark, and are often retained and enriched in agricultural and food processing by-products [4,5]. Because polyphenols are so widely distributed, they are often the primary target for resource recycling and high-value utilization. Therefore, current research has increasingly focused on green and efficient extraction and separation processes for plant byproducts. These processes are important for recovering bioactive compounds and adding value to plant residues [6,7].

Previous research indicates that polyphenols typically exist in bound form within the lignocellulose cell wall network. Therefore, how to efficiently and selectively release polyphenols from complex substrates is a key aspect of many scholars’ research into their potential value [8,9]. It has been widely reported that alkaline treatment can destroy the lignin and hemicellulose network and cleave ester bond linkages, which has a significant promoting effect on the release of bound polyphenols. However, excessively alkaline conditions can cause some active ingredients inside the plant to be broken down, thereby reducing the selectivity of polyphenol extraction [10,11]. Therefore, the polyphenol extraction process not only aims at the endpoint yield, but also needs to clarify the impact of different process steps on the transfer resistance, release pathways and potential component stability during the extraction process. Compared with alkaline extraction, acid treatment mainly promotes the migration of target components by hydrolyzing glycosidic bonds and weakening the cell wall barrier. However, its ability to release phenols bound by ester bonds is limited, and achieving a balance between final yield and compound integrity may be difficult [12]. To address this problem, acid pretreatment followed by alkaline extraction is becoming more common. In this process, the cell wall is first broken down selectively, then polyphenols are released, and the yield is usually higher than with a single stage [13,14,15]. It has been reported that pyruvic acid can weaken cell wall barriers under mild conditions and mitigate potential damage to polyphenol structure as an alternative to inorganic acids [16], indicating promising application potential. However, the endpoint extraction yield alone could not reveal the true contribution of pretreatment to the disassembly of the cell wall structure. Therefore, a thorough kinetic analysis of the extraction process was required. For example, Qin et al. [17] combined extraction kinetics, molecular dynamics simulations, and theoretical calculations. Their results showed that polyphenol release was controlled not only by the apparent extraction rate but also by interactions between solvent and solute, as well as mass transfer enhancement mechanisms. Xia et al. [18] developed a kinetic model coupled with diffusion for the acid pretreatment of wheat straw, enabling quantitative description of the simultaneous changes in mass transfer and reaction behavior within the particles. Shen et al. [19] studied the extraction of chlorogenic acid from Eucommia ulmoides leaves using a second-order kinetic approach and Fick’s second law. They calculated the effective diffusion coefficient, mass transfer coefficient and Biot number, and suggested that the extraction rate was mainly controlled by intraparticle diffusion. In summary, sequential PA NaOH extraction improved the release of bound polyphenols from lignocellulosic matrices. The kinetic analysis further showed that the extraction resistance was not controlled by a single step, but changed during the release process. However, previous studies have not fully clarified how structural changes are related to mass transfer behavior at different extraction stages. Therefore, it is necessary to conduct a systematic analysis of this process using staged kinetic modeling [20,21,22].

**Figure 1:**
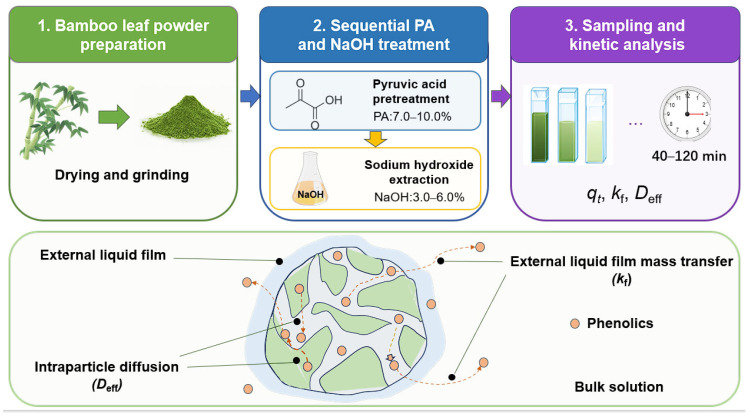
Schematic illustration of the experimental workflow and mass transfer process during sequential PA pretreatment and NaOH extraction of bamboo leaf phenolics.

This study utilized bamboo leaves as the raw material from forestry by-products and adopted an acid-base sequential extraction strategy to investigate the kinetics of polyphenol transport. Using PA concentration and NaOH concentration as variables, a time-course experiment based on a full factorial design was conducted under uniform particle size and solid-to-liquid ratio conditions, and a standardized dataset was constructed. The Folin Ciocalteu method was used to quantitatively characterize the polyphenol yield. Based on the normalized conversion rate, a kinetic framework with two stages and two parameters was constructed. In the early stage, the linear driving force model was used to describe external liquid film mass transfer, whereas in the later stage, the spherical Fickian diffusion model was used to describe intraparticle diffusion. The external liquid film mass transfer coefficient *k*_f_ and the effective diffusion coefficient *D*_eff_ were used to evaluate the relative contributions of external liquid film transfer and intraparticle diffusion during extraction. The results indicated that the extraction resistance changed during the release process rather than being controlled by a single step. The conceptual framework of this study is shown in Figure 1. The results supported the use of the kinetic model for interpreting the release behavior of total phenolics during PA NaOH sequential extraction. The analysis also described the stage-dependent transport characteristics of the extraction process and provided a kinetic reference for optimizing bamboo leaf polyphenol extraction.

## 2. Materials and Methods

### 2.1. Materials

Fresh bamboo leaves collected from Chengdu (Sichuan, China) were dried, crushed, and ground into powder with a particle size of 40–60 mesh. The following analytical reagent grade (AR) chemicals were used: PA ≥ 98%, NaOH ≥ 98%, Folin Ciocalteu phenol reagent, and gallic acid ≥ 98.0% for the standard curve. We purchased all other chemicals and reagents of analytical grade from Aladdin, Shanghai, China.

### 2.2. Experimental Design and Kinetic Data Acquisition

We mixed 5 g of bamboo leaf powder with pyruvic acid solution at a solid-to-liquid ratio of 1:10. The mixture was then heated to 60–80 °C for acid pretreatment. After the specified time, the mixture was cooled rapidly and the solid residue was collected by filtration. This solid was washed with distilled water until neutral, which stopped the acid reaction and removed soluble impurities. The treated residue was then mixed with aqueous NaOH at the same ratio (1:10, solid/liquid) and extracted at 25–45 °C for 40–120 min. After cooling and filtration, the filtrate was saved.

The alkaline extract was neutralized to pH 7.0 using dilute hydrochloric acid. It was then centrifuged at 6000 rpm for 10 min (CenLee 2050R, Xiang Li, Changsha, China). The supernatant was filtered through a 0.45 μm membrane to obtain a clear extract. Total polyphenol content was determined at 765 nm with the Folin Ciocalteu method [23,24]. The polyphenol yield expressed in terms of gallic acid equivalent (mg GAE/g) was calculated from the standard curve [25].

PA concentration and NaOH concentration were selected as the two independent variables. PA concentration was set at 7.0, 8.0, 9.0 and 10.0%, while NaOH concentration was set at 3.0, 4.0, 5.0 and 6.0%. The combination of these two factors generated sixteen extraction groups, labeled as G1 to G16, as shown in Table 1.

For kinetic data acquisition, each extraction group was sampled at 40, 60, 80, 100 and 120 min. The total phenolic yield at each sampling time was determined using the Folin Ciocalteu method and expressed as mg GAE/g dry biomass. The obtained yield at each time point was defined as *q_t_*, and the *q_t_* values at the five sampling times were used to construct the kinetic curve for each extraction group.

### 2.3. Polyphenol Dissolution Kinetics Models

To eliminate the influence of the differences in the initial adsorption amount (*q*_0_) and the equilibrium adsorption amount (*q_∞_*) under different experimental conditions on the kinetic analysis, the absolute adsorption amount data were first converted into the dimensionless normalized fractional conversion rate *F*(*t*) [26], which is defined as:

$$F(t)=\frac{q_{t}-q_{0}}{q_{\infty }-q_{0}}$$

where *q_t_* (mg GAE/g) is the total phenolic yield at time *t* (min), *q*_0_ is the reference yield at the first measured point, 40 min. It is used only to normalize the observed time course and does not mean that 40 min is the physical extraction origin *t* = 0.

The equilibrium yield *q*_∞_ was estimated by fitting the experimental *q*_t_ data over time to a first-order saturation model using nonlinear least squares regression:

$$q(t)=q_{\infty }-(q_{\infty }-q_{0})exp[-k(t-t_{0})]$$

The value of *F*(*t*) ranges from 0 to 1 within the normalized observed curve. F(t) = 0 corresponds to the first measured reference point at 40 min, whereas F(t) approaches 1 as *q*_t_ approaches *q*_∞_. Thus, F(t) describes release progression within the measured time course and supports comparison among conditions.

Normalized conversion was first examined to identify the portion of the release curve in which *ln* [1 − *F*(*t*)] showed an approximately linear change with time. Because the discrete F(t) values were unevenly distributed among the five sampling points, a single rigid F(t) cutoff would have produced different numbers of fitted observations among conditions. For consistent parameter estimation across all 16 groups, the observations at 40, 60, and 80 min were therefore used as the LDF fitting window [27]. This operational window describes the first measured portion of the curve and does not assign external liquid film transfer to an exclusive physical stage. The integrated LDF form is given as:

$$ln[1-F(t)]=-k_{f}\cdot t+b$$

where *k*_f_ is the apparent external liquid film mass transfer coefficient, expressed in min^−1^, and *b* is the fitting intercept.

For each experimental group, ln [1 − F(t)] at 40, 60, and 80 min was regressed against time. The negative slope of the fitted line was used to estimate the apparent external liquid film mass transfer coefficient *k*_f_.

The Crank spherical diffusion model was applied to the observations at 80, 100, and 120 min, which form the Crank fitting window used to estimate *D*_eff_. The 80 min observation was included in both fitting windows as a shared transition point. This overlap is consistent with simultaneous external liquid film transfer and intraparticle diffusion and does not assign either process to an exclusive time period. Bamboo leaf particles were approximated as homogeneous spheres with constant surface concentration as the boundary condition. The first-term approximation of the Crank model is expressed as follows:

$$1-F(t)\approx \frac{6}{\pi^{2}}exp\left(-\frac{\pi^{2}D_{eff}}{r^{2}}t\right)$$

where *D*_eff_ is the effective diffusion coefficient of phenolics within bamboo leaf particles, expressed in m^2^/s, and *r* is the equivalent particle radius, expressed in m.

To linearize the equation, we took the natural logarithm of both sides, giving:

$$ln[1-F(t)]=ln\left(\frac{6}{\pi^{2}}\right)-\left(\frac{\pi^{2}D_{eff}}{r^{2}}\right)t$$

The theoretical intercept is *A* = *ln*(6/*π*^2^) ≈ −0.4971, and the slope is *B* = *π*^2^*D*_eff_/*r*^2^. Linear regression of *ln* [1 − *F*(*t*)] against time was performed using the observations at 80, 100, and 120 min. The fitted slope B was then used to calculate *D*_eff_.

Based on the fitted slope B, expressed in s^−1^ after conversion from min−1, and the known particle radius r, the effective diffusion coefficient was calculated as:

$$D_{eff}=\frac{B\cdot r^{2}}{\pi^{2}}$$

The bamboo powder was 40 to 60 mesh, so we took its average radius as 0.326 mm. Therefore, the equivalent radius *r* = 1.63× 10^−4^ m.

Model Diagnosis: The rationality of the spherical diffusion approximation was evaluated from the intercept deviation Δa = |Afit − Atheory|, where Atheory = −0.4971.

### 2.4. Model Selection for Kinetic Analysis

To avoid relying only on a predefined two-stage mass transfer model, several commonly used kinetic models were further used to fit the complete extraction curves. The fitting was performed directly using the original *q_t_* values at 40, 60, 80, 100 and 120 min under each PA and NaOH condition. The normalized conversion *F*(*t*) was not used in this model selection step, so that the comparison was independent of the subsequent segmented mass transfer analysis.

Five kinetic models were selected for comparison, including the pseudo first order model, pseudo second order model, Peleg model, Weibull model and Elovich model. The equations used for fitting are listed in Table 2. For each PA and NaOH combination, the complete *q_t_* curve was fitted separately using each model. Nonlinear regression was performed by minimizing the sum of squared residuals between the experimental *q*_t_ values and the fitted *q_t_* values.

The fitting quality was evaluated using the coefficient of determination R^2^, root mean square error RMSE, mean absolute error MAE, corrected Akaike information criterion AICc and Bayesian information criterion BIC. R^2^ was used to describe the agreement between the experimental and fitted values, while RMSE and MAE were used to evaluate the fitting error. Because models with more adjustable parameters may show lower residual error without better reliability, AICc and BIC were also used to compare model performance after considering model complexity.

The statistical indicators were calculated as follows:

$$RMSE=\sqrt{\frac{1}{n}\sum_{i=1}^{n}(q_{exp,i}-q_{fit,i}{)}^{2}}$$

$$MAE=\frac{1}{n}\sum_{i=1}^{n}|q_{exp,i}-q_{fit,i}|$$

$$AICc=nln\left(\frac{SSE}{n}\right)+2k+\frac{2k(k+1)}{n-k-1}$$

$$BIC=nln\left(\frac{SSE}{n}\right)+kln(n)$$

where *q*_erp,i_ and *q*_fit,i_ are the experimental and fitted total phenolic yields at sampling point iii, respectively; *n* is the number of data points used for fitting; *k* is the number of fitted model parameters; and SSE is the sum of squared errors. Lower RMSE, MAE, AICc and BIC values indicate better model performance. For each experimental condition, the model with the lowest AICc value was regarded as the most parsimonious model for describing the complete extraction curve.

### 2.5. Determination of Mass Transfer Control Steps

To describe the rate control stage of the extraction process quantitatively, the characteristic time scale ratio of external liquid film mass transfer and intraparticle diffusion, *τ*_m_/*τ*_d_, was introduced as a criterion. Here, *τ*_m_ = 1/*k*_f_ represented the time scale of external liquid film mass transfer based on the linear driving force model and *τ*_d_ = *r*^2^/(*π*^2^*D*_eff_) represented the first-order characteristic timescale of intraparticle diffusion in spherical particles [28]. The former originated from the first-order response form of the LDF model, while the latter originated from the first characteristic time of the Crank solution of Fick’s second law in spherical coordinates [29].

Studies usually distinguish external film mass transfer from intraparticle diffusion via characteristic time ratios, Biot numbers, or mixed models. To make comparisons clearer, we set *τ*_m_/*τ*_d_ < 0.5 as intraparticle diffusion control, 0.5 to 2 as mixed control, and > 2 as external liquid film mass transfer control. At the same time, the threshold was not used as an absolute boundary, but as a way to identify which step was more likely to slow down the overall extraction process.

### 2.6. Predictive Validation of the Kinetic Framework

To evaluate the predictive ability of the kinetic framework, both internal validation and independent experimental validation were carried out. The original sixteen PA and NaOH combinations were used as the training dataset. The validation procedures were performed without using the validation data during model fitting.

For internal validation, leave-one-condition-out validation was used. In each round, one PA and NaOH combination was removed from the dataset, and the remaining fifteen combinations were used to establish the prediction model. The removed condition was then predicted using the model trained from the remaining data. This procedure was repeated until each of the sixteen original conditions had been used once as the validation condition. The predicted *q_t_* values were compared with the corresponding experimental *q_t_* values at 40, 60, 80, 100 and 120 min. Curve RMSE, curve MAE and the absolute relative error of *q*_120_ were calculated for each validation round.

For independent experimental validation, two additional PA and NaOH combinations that were not included in the original factorial design were selected. The condition of 7.0% PA and 4.5% NaOH was used as an interpolation validation point within the original design range. The condition of 11.0% PA and 7.0% NaOH was used as an extrapolative trend test outside the original design range. These two validation experiments were performed using the same extraction procedure, sampling times and total phenolic content determination method as the original kinetic experiments. The measured *q_t_* values from these two conditions were used only for validation and were not included in model fitting.

To generate predicted *q_t_* values for the validation conditions, the Peleg equation was used as the predictive curve equation:

$$q_{t}=\frac{t}{K_{1}+K_{2}t}$$

First, the original sixteen experimental conditions were fitted using the Peleg equation to obtain *K*_1_ and *K*_2_ for each condition. Then, *K*_1_ and *K*_2_ were separately fitted as functions of PA and NaOH concentration using a second-order response equation:

$$K_{i}=b_{0}+b_{1}PA+b_{2}NaOH+b_{12}PA\times NaOH+b_{11}PA^{2}+b_{22}NaOH^{2}$$

where *K*_i_ represents either *K*_1_ or *K*_2_, and *b*_0_, *b*_1_, *b*_2_, *b*_12_, *b*_11_ and b_22_ are fitted coefficients. The PA and NaOH concentrations of the validation conditions were then substituted into the response equations to obtain the predicted *K*_1_ and *K*_2_ values. These predicted parameters were finally substituted into the Peleg equation to calculate the predicted *q_t_* values at each sampling time.

The prediction error was evaluated using curve RMSE, curve MAE, residuals and absolute relative error. The residual was calculated as:

$$Residual=q_{exp}-q_{pred}$$

The absolute relative error was calculated as:

$$Absolute\;\mathrm{relative}\;\mathrm{error}\;(\%)=\left|\frac{q_{exp}-q_{pred}}{q_{pred}}\right|\times 100$$

where *q*_exp_ is the measured polyphenol yield and *q*_pred_ is the predicted polyphenol yield. For the endpoint yield, the absolute relative error of *q*_120_ was calculated using the same equation. The internal validation results and the independent validation results were then used to assess the predictive performance of the kinetic framework within and beyond the original concentration range.

## 3. Results and Discussion

### 3.1. Effect of Pyruvic Acid Concentration on Polyphenol Dissolution

**Figure 2:**
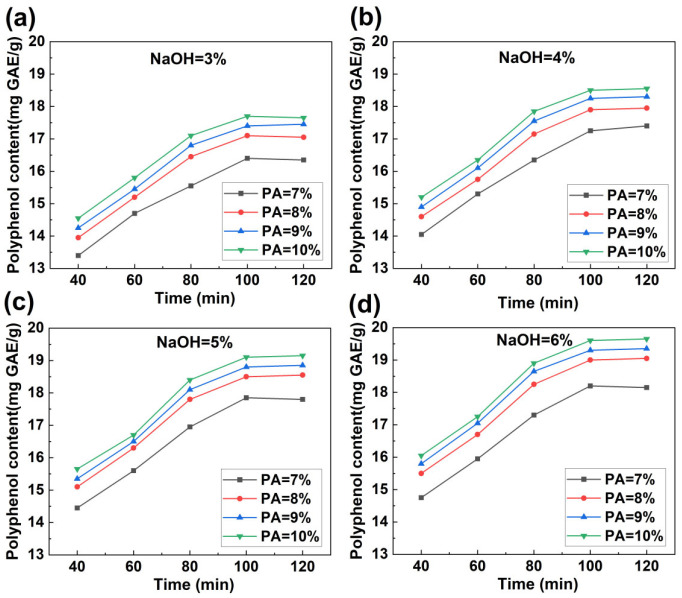
Kinetic curves for total phenolic yield at different PA concentrations. Panels (**a**–**d**) correspond to NaOH concentrations of 3.0, 4.0, 5.0, and 6.0%, respectively.

After acid extraction, the alkali extraction stage of polyphenols was affected by the alkali concentration and reaction time, which directly influence the depolymerization behavior and the transfer of components in the lignocellulose matrix [30]. To assess these effects, the content of polyphenols released with increasing hydrolysis time was studied at different concentrations of NaOH (3.0%, 4.0%, 5.0%, and 6.0%) and different reaction times (40, 60, 80, 100, and 120 min), as shown in Figure 2. The complete data for this set of experiments are shown in Table S1.

In the extraction of plant fiber components, the final yield is affected by the concentrations of PA and NaOH, and the reactivity of PA is also affected by the concentration of NaOH [31].

At lower alkalinity (3.0%, Figure 2a), the promoting effect of PA on polyphenols was mainly reflected in the early and middle stages. Even after increasing the PA concentration from 7.0% to 10.0%, the final yield only increased by 7.9%, and the difference in the curves was small in the later stages of extraction, indicating that the structural improvement brought about by PA pretreatment cannot be fully realized at lower alkalinity concentrations. At 4.0% NaOH (Figure 2b), the effect of PA became more evident. The extraction curves at different PA concentrations showed a similar upward trend throughout the extraction period, and the final total phenolic yield increased by 6.6%. This suggests that PA provided a more sustained improvement in extraction under this NaOH condition. However, under the condition of 5.0% NaOH (Figure 2c), the effect of PA showed a turning point. Among them, a PA concentration of 8.0% showed good kinetic characteristics, with a final yield of 18.55 mg/g. However, when the PA concentration was further increased to 9.0% and 10.0%, the growth rate decreased, indicating that further increasing the PA concentration had little effect on improving the extraction performance of polyphenols. At the highest alkali concentration (6.0%, Figure 2d), the advantage of PA concentration was stable but showed a decreasing trend, with relatively balanced curve spacing. Nevertheless, PA still showed good extraction efficiency in the 60–100 min range.

In summary, 8.0% PA showed a favorable kinetic performance in this experimental group, especially when the NaOH concentration was 5.0% or higher. This result suggests that the extraction performance depends on the combined PA and NaOH treatment conditions rather than on PA concentration alone. Furthermore, as the PA concentration further increased, the improvement in extraction yield became less pronounced, suggesting that an optimal acid treatment intensity exists in this reaction system. This finding indicates that the resistance encountered during PA NaOH sequential extraction is not controlled by a single step, which is important for understanding and optimizing the recovery of phenolic-rich extracts from bamboo leaves. Using excessively high acid concentrations may have a limited effect on further improving extraction and may also cause nonselective degradation of biomass components, which can reduce the recovery of phenolic compounds.

### 3.2. Effect of NaOH Concentration on Polyphenol Dissolution

**Figure 3:**
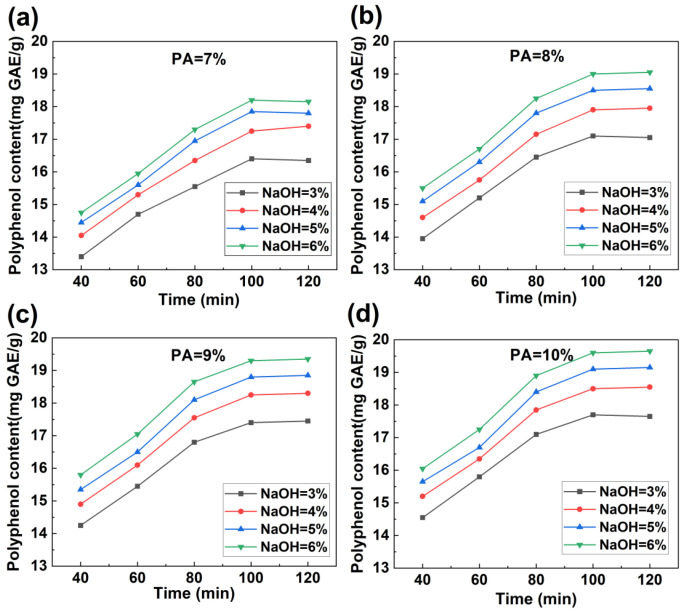
Kinetic curves for total phenolic yield at different NaOH concentrations. Panels (**a**–**d**) correspond to PA concentrations of 7.0, 8.0, 9.0, and 10.0%, respectively.

Figure 3 shows how the effect of NaOH concentration on the extraction process varied under different PA pretreatment conditions. At a PA concentration of 7.0% (Figure 3a), when the NaOH concentration increased from 3.0% to 6.0%, the final polyphenol yield increased from 16.35 mg/g to 18.15 mg/g, with an absolute increase of 1.80 mg/g. The curves show approximately parallel growth over different time periods, indicating that the promoting effect of NaOH is relatively uniform under the current conditions, and that there is a certain linear relationship in this system. At 8.0% PA (Figure 3b), the effect of NaOH concentration on total phenolic yield became more evident. The final yield increased from 17.05 to 19.05 mg/g under the same NaOH concentration change, giving an absolute increase of 2.00 mg/g. It is notable that the curves with NaOH concentrations of 4.0% and 5.0% showed a significantly increased slope from 60 to 80 min, indicating that under these conditions, the promoting effect of PA pretreatment on subsequent alkaline extraction was the most prominent. This result is consistent with previous studies suggesting that PA pretreatment can promote hemicellulose separation, reduce lignin recondensation, and improve substrate accessibility [16]. When the PA concentration increased to 9.0% (Figure 3c), the increase in the endpoint brought about by the change in NaOH concentration decreased slightly to 1.90 mg/g, and the curves of 5.0% and 6.0% NaOH tended to be close at the high position, indicating that at higher PA conditions, the marginal effect of further increasing NaOH concentration weakened, and the system tended to be saturated.

At 10.0% PA (Figure 3d), the final total phenolic yield remained at a relatively high level, although the improvement was less pronounced than that observed at 8.0% PA. It is noted that the curve for 3.0% NaOH was significantly lower than that for high NaOH concentrations. This indicated that in the system after strong acid treatment, the structural breakdown provided by PA at lower alkali concentrations cannot be fully converted into the final extraction yield. Furthermore, the difference in final yield between higher NaOH concentrations was not significant, suggesting that excessively high acid concentrations did not provide the expected additional benefits. This trend is consistent with general observations in lignocellulosic biomass pretreatment, where a suitable treatment intensity can improve matrix disruption and facilitate the release of target compounds. However, excessive treatment intensity may cause nonselective degradation of biomass components and secondary condensation reactions, resulting in limited further improvement in extraction yield [32,33,34,35,36].

Therefore, NaOH was the dominant factor determining the final yield level, and its effectiveness depended on the PA pretreatment concentration. When the PA concentration was lower or higher than 8.0%, further increasing the NaOH concentration produced only limited improvement in extraction performance. This suggests that NaOH affected the extraction process in relation to the PA treatment condition, rather than acting as an independent factor.

### 3.3. Kinetic Model Evaluation and Mass Transfer Analysis

Five commonly used kinetic models were first fitted to the complete *q_t_* curves under the 16 combinations of PA and NaOH concentrations. These models included the pseudo first order model, pseudo second order model, Peleg model, Weibull model, and Elovich model. The experimental *q_t_* values at 40, 60, 80, 100, and 120 min were used directly for fitting. The normalized conversion fraction *F*(*t*) was not used in this step. The equations and fitted parameters are listed in Table 2, while the statistical data are provided in Table S2.

The comparison did not identify a model that performed consistently well under all 16 conditions. Although the five models generally reproduced the increase in total phenolic yield with extraction time, their fitting quality varied among the experimental conditions. In some cases, a relatively high R^2^ was accompanied by larger RMSE or MAE values. The model rankings also changed when AICc and BIC were considered. Therefore, no single empirical model could provide a consistently reliable description of all the extraction curves. These results suggest that the change in the extraction rate over time cannot be fully represented by one empirical equation. The five models were therefore retained for comparison, but they were not used to determine the main mass transfer resistance or to calculate the mass transfer coefficients.

The extraction of phenolic compounds from plant materials generally shows a relatively rapid increase followed by a gradual approach to equilibrium. Previous studies have used different kinetic equations and diffusion models to describe this change in extraction behavior [37,38,39]. In this study, F(t) was used to evaluate normalized release progression, and the LDF and Crank models were applied to two overlapping fitting windows with a consistent number of observations. External liquid film transfer and intraparticle diffusion were considered to occur at the same time. The fitting windows are used only for parameter estimation and do not represent two physically separate processes.

**Figure 4:**
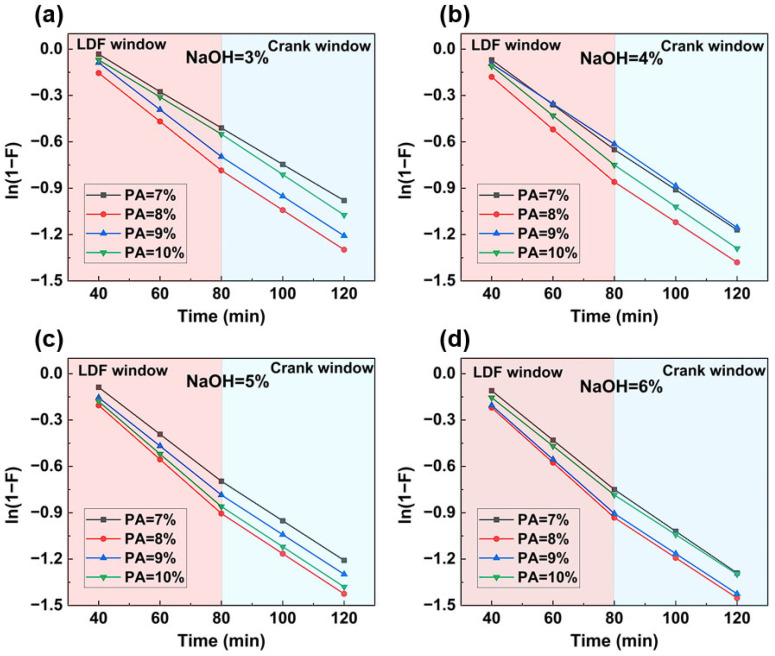
Linearized LDF and Crank fits used to estimate *k*_f_ and *D*_eff_. Panels (**a**–**d**) correspond to NaOH concentrations of 3.0%, 4.0%, 5.0%, and 6.0%, respectively, with four PA concentrations in each panel. The left and right shaded regions indicate the LDF and Crank fitting windows, respectively.

Figure 4 shows the linear fitting results under the 16 experimental conditions. For the LDF fitting window, the observations at 40, 60, and 80 min gave a linear relationship between *ln* [1 − *F*(*t*)] and extraction time, with R^2^ values above 0.95 for all conditions. The slope was used to calculate the apparent external liquid film mass transfer coefficient *k*_f_. The F(t) values at the same sampling time varied among PA and NaOH conditions, so F(t) was used to evaluate release progression rather than as a rigid universal cutoff.

For the Crank fitting window, the observations at 80, 100, and 120 min also gave linear relationships, with *R*^2^ values above 0.97. The fitted intercepts were close to *ln*(6/*π*^2^), approximately −0.497, and the mean absolute deviation from the theoretical intercept was below 0.03. These results support the use of the spherical diffusion approximation to estimate *D*_eff_, but they do not mean that intraparticle diffusion was the only transport process in this window. The process associated with the greater relative resistance was identified from the characteristic time ratio. The complete fitting results are presented in Table S3.

### 3.4. Effects of PA and NaOH Concentrations on Mass Transfer Parameters

**Figure 5:**
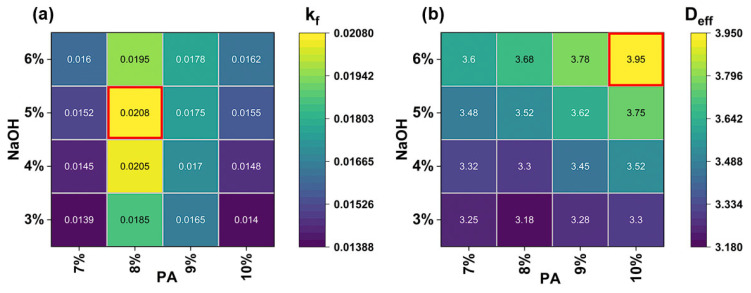
Heat maps of the apparent external liquid film mass transfer coefficient *k*_f_ and the effective intraparticle diffusion coefficient *D*_eff_ under different PA and NaOH concentrations. Panel (**a**) shows *k*_f_, and panel (**b**) shows *D*_eff_. The red boxes indicate the maximum (*k*_f_) and (*D*_eff_) values under the tested conditions, occurring at 8.0% PA with 5.0% NaOH and 10.0% PA with 6.0% NaOH, respectively.

Figure 5 shows the changes in the apparent external liquid film mass transfer coefficient *k*_f_ and the effective intraparticle diffusion coefficient *D*_eff_ under the 16 combinations of PA and NaOH concentrations. The corresponding values and the total phenolic yields at 120 min are listed in Table 3. The endpoint yield and the two transport parameters were compared together because they describe different aspects of the extraction process [40].

As shown in Figure 5a, *k*_f_ first increased and then decreased with increasing PA concentration when the NaOH concentration was fixed. Higher *k*_f_ values were mainly observed at 8.0% PA, with a local maximum at 8.0% PA and 5.0% NaOH. Changing the NaOH concentration affected the value of *k*_f_, but did not clearly change the PA concentration at which the maximum occurred. The statistical analysis showed that both PA and NaOH had significant effects on *k*_f_, with *p* < 0.001 for PA and *p* = 0.002 for NaOH. However, increasing the PA concentration above 8.0% did not further increase *k*_f_. This result indicates that a higher PA concentration did not continuously improve external liquid film transfer. Similar nonlinear changes have been reported at high acid treatment intensities, where additional chemical changes and competing reactions may reduce the expected treatment efficiency [41,42].

The variation in *D*_eff_ followed a different pattern. As shown in Figure 5b, *D*_eff_ increased continuously with the NaOH concentration at each PA level. The highest *D*_eff_ was obtained at 10.0% PA and 6.0% NaOH. The statistical results showed significant effects of both NaOH and PA on *D*_eff_, with *p* < 0.001 for NaOH and *p* = 0.0005 for PA. In contrast to the change in *k*_f_, no turning point was observed in the response of *D*_eff_ to NaOH within the tested concentration range. Effective diffusion may be influenced by resistance at the solid-liquid interface, the accessibility of transport pathways, and interactions between the extracted compounds and the surrounding matrix [43,44]. Increasing the NaOH concentration may also change the ionization state and liquid-phase behavior of phenolic compounds, which can favor their migration from the solid matrix into the extraction solution [45,46]. The fitted *D*_eff_ values reflect the combined influence of these factors, but cannot separate their individual contributions.

The endpoint total phenolic yield increased with both PA and NaOH concentrations. The highest yield was 19.65 ± 0.24 mg GAE/g at 10.0% PA and 6.0% NaOH. The effects of PA and NaOH on the endpoint yield were both significant, with *p* < 0.001, and their interaction was also significant, with *p* = 0.028. The complete statistical results are provided in Table S4. The condition with the highest endpoint yield and *D*_eff_ was different from the condition associated with the local maximum of *k*_f_. For example, 8.0% PA and 5.0% NaOH gave a relatively high *k*_f_, but its endpoint yield was 18.55 ± 0.26 mg GAE/g, which was lower than the maximum value. Therefore, the condition selected later for process optimization should be regarded as a balanced condition rather than the condition giving the highest endpoint yield.

Overall, PA concentration was associated with the increase and subsequent decrease in *k*_f_, while NaOH concentration showed a continuous positive relationship with *D*_eff_. These different responses indicate that PA and NaOH affected the two transport parameters in different ways. However, the parameter trends alone cannot determine which transport process produced the greater resistance.

### 3.5. Rate Control Analysis Based on Characteristic Time Scales

**Figure 6:**
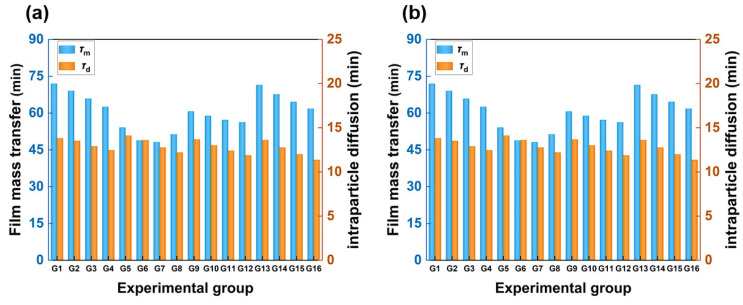
Characteristic time comparison for external liquid film mass transfer and intraparticle diffusion under the 16 treatment conditions. Panel (**a**) compares *τ*_m_ and *τ*_d_, and panel (**b**) shows the ratio *τ*_m_/*τ*_d_. The dashed line at 2 marks the relative resistance criterion used in this study.

The characteristic time scales of external liquid film transfer and intraparticle diffusion were compared to determine their relative contributions to the extraction resistance. The characteristic time of external liquid film transfer was expressed as *τ*_m_ = 1/*k*_f_, while the characteristic time of intraparticle diffusion was expressed as *τ*_d_ = *r*^2^/(*π*^2^*D*_eff_). A longer characteristic time represents a slower transport process under the same experimental condition. Figure 6a compares *τ*_m_ and *τ*_d_, and Figure 6b presents their ratio.

The value of *τ*_m_/*τ*_d_ ranged from 2.85 to 5.27 across the 16 experimental conditions. The lowest value of 2.85 was obtained at 8.0% PA and 3.0% NaOH, while the highest value of 5.27 was obtained at 10.0% PA and 6.0% NaOH. All ratios exceeded the criterion of 2 used in this study. This means that *τ*_m_ was more than twice *τ*_d_ under all tested conditions. According to the criterion used in this study, external liquid film transfer produced a greater relative resistance than intraparticle diffusion.

At each NaOH concentration, the values of *τ*_m_/*τ*_d_ were generally lower at 8.0% PA than at the other PA concentrations. This result agrees with the higher *k*_f_ values observed at 8.0% PA. The increase in *k*_f_ reduced *τ*_m_, which decreased the difference between the two characteristic time scales. When the NaOH concentration increased, *D*_eff_ also increased and *τ*_d_ became shorter. As a result, the ratio of *τ*_m_ to *τ*_d_ generally increased. This indicates that increasing the NaOH concentration accelerated intraparticle diffusion more clearly than external liquid film transfer. External liquid film resistance therefore became more evident relative to intraparticle diffusion at higher NaOH concentrations.

The ratio above 2 does not mean that intraparticle diffusion was absent. It also does not indicate that external liquid film transfer and intraparticle diffusion occurred as two completely separate steps. Both transport processes can occur at the same time, while their relative resistances change with the extraction conditions [47]. The criterion of 2 was used here for comparison among the experimental conditions and should not be regarded as a universal physical boundary.

The main fitting and statistical results are summarized in Table 4. The characteristic time analysis identifies the transport process that produced the greater relative resistance, but it does not explain the physical or chemical origin of the changes in *k*_f_ and *D*_eff_. Kinetic parameters alone cannot determine whether these changes were related to the solid surface, the internal morphology, or the chemical environment during extraction. SEM and FTIR analyses were therefore used to examine whether the observed mass transfer trends were accompanied by changes in surface morphology and chemical environment.

### 3.6. Structural Evidence and Model Validation

**Figure 7:**
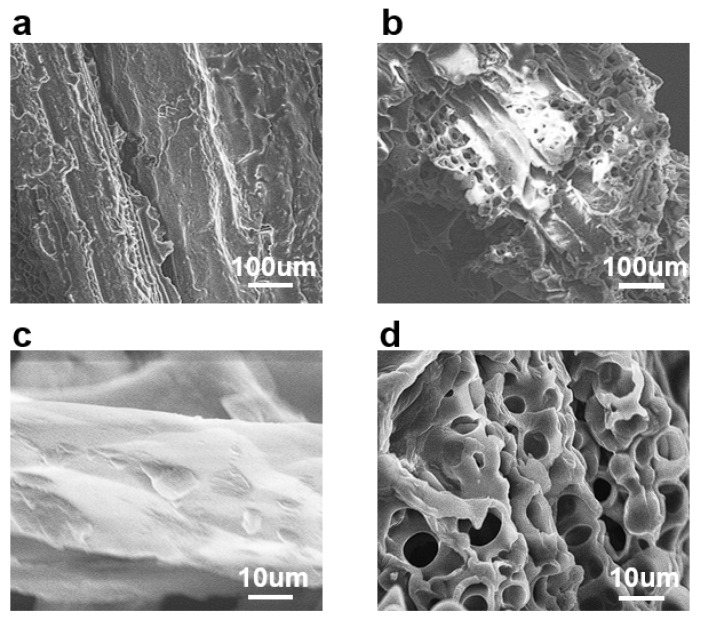
SEM images of bamboo leaf samples before and after PA and NaOH treatment. Panels (**a**,**c**) show untreated samples, and panels (**b**,**d**) show the treated samples.

SEM was used to examine the surface morphology of the bamboo leaf samples before and after treatment. As shown in Figure 7, the untreated sample had a relatively compact and continuous surface. After PA pretreatment followed by NaOH extraction, the surface became rougher, and irregular openings and local fractures were observed. These changes increased the exposed surface and may have made solvent penetration and compound release easier. Similar changes in surface morphology and material accessibility have been reported after acid treatment of lignocellulosic materials [48,49,50]. The SEM results are therefore consistent with the changes in the fitted mass transfer parameters, although they cannot separately determine the effects of PA and NaOH.

**Figure 8:**
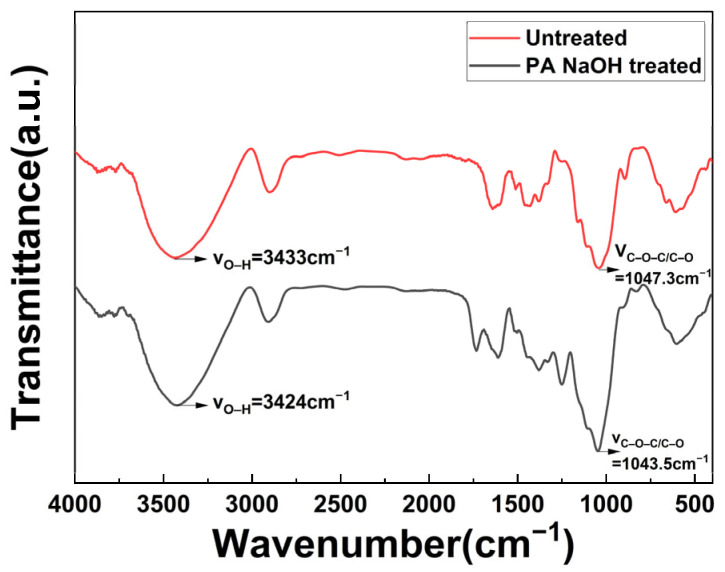
FTIR spectra of bamboo leaf samples before and after sequential PA pretreatment and NaOH extraction. The marked bands assigned to OH, C–O, aromatic structures, and C–O–C.

The FTIR spectra are shown in Figure 8. Both samples retained the main bands assigned to O–H, C-–O, aromatic structures, and C–O–C bonds. The C–O–C related band shifted slightly from approximately 1047 cm^−1^ before treatment to 1044 cm^−1^ after treatment. This shift indicates a change in the local chemical environment rather than the formation of a new functional group. Similar changes in oxygen-containing groups have been observed in biomass treatment systems involving acidic or alkaline conditions [51,52]. The SEM and FTIR results support the structural interpretation of the changes in *k*_f_ and *D*_eff_, while the identification of the main transport resistance remains based on the characteristic time scale analysis.

Since none of the empirical models performed consistently best under all experimental conditions, the Peleg equation was used as a practical equation for curve prediction. Leave-one-condition-out validation gave a mean RMSE of 0.179 mg GAE/g and a mean MAE of 0.150 mg GAE/g. The mean absolute relative error of *q*_120_ was 0.71%, with individual values ranging from 0.03% to 2.11%. The detailed validation results for the 16 conditions are provided in Table S5.

The results for the two additional validation conditions are summarized in Table 5. At 7.0% PA and 4.5% NaOH, the measured and predicted *q*_120_ values were 17.57 and 17.60 mg GAE/g, respectively. The RMSE was 0.040 mg GAE/g, the MAE was 0.032 mg GAE/g, and the endpoint error was 0.15%. At 11.0% PA and 7.0% NaOH, the measured and predicted *q*_120_ values were 19.94 and 20.20 mg GAE/g. The corresponding RMSE, MAE, and endpoint error were 0.171 mg GAE/g, 0.142 mg GAE/g, and 1.27%, respectively. The error was higher at conditions above the original concentration range, but the predicted curve still followed the experimental trend. These results show that the model provided reliable predictions within the tested range, while predictions beyond this range should be interpreted with caution.

The Folin Ciocalteu assay quantified the total phenolic fraction but did not distinguish individual phenolic compounds. Accordingly, the fitted parameters describe total phenolic release rather than compound-specific transport. Future studies should combine chromatographic analysis, such as HPLC DAD or LC MS, with the kinetic framework to determine whether individual phenolics show different release behavior.

### 3.7. Process Condition Selection and Temperature Dependence

**Figure 9:**
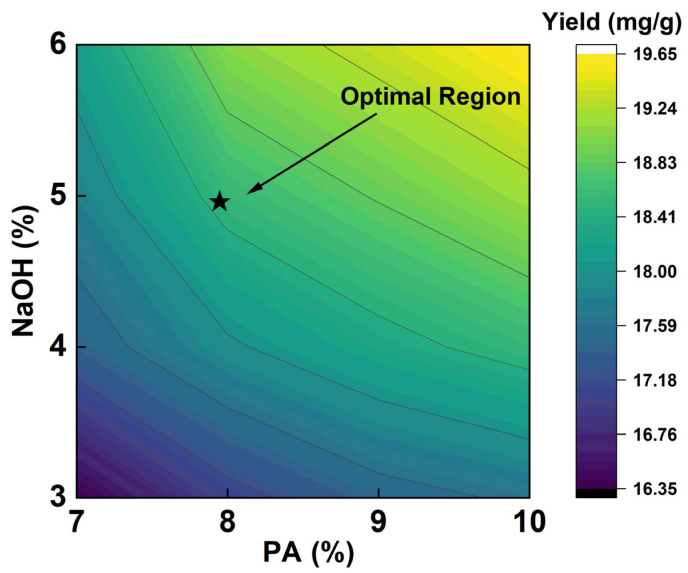
Response surface contour for total phenolic yield at 120 min as a function of PA and NaOH concentrations.

Figure 9 shows the effects of PA and NaOH concentrations on the total phenolic yield at 120 min. The response increased from the lower left region to the upper right region, indicating that both PA and NaOH promoted the endpoint yield within the tested concentration range. The region with relatively high yields was mainly located between 8.0% and 10.0% PA and between 5.0% and 6.0% NaOH. The highest experimental yield was 19.65 ± 0.24 mg GAE/g at 10.0% PA and 6.0% NaOH.

Although 8.0% PA and 5.0% NaOH did not give the highest endpoint yield, this condition was located within the high response region and produced 18.55 ± 0.26 mg GAE/g. This value was approximately 94.4% of the maximum yield. It was also associated with a high *k*_f_, a *D*_eff_ value of 4.20 × 10^−12^ m^2^/s, and a relatively low *τ*_m_/*τ*_d_ value of 3.02. Increasing the concentrations to 10.0% PA and 6.0% NaOH produced only a moderate increase in yield while requiring more acid and alkali. Therefore, 8.0% PA and 5.0% NaOH were selected as a balanced operating condition rather than the condition giving the absolute maximum yield.

**Figure 10:**
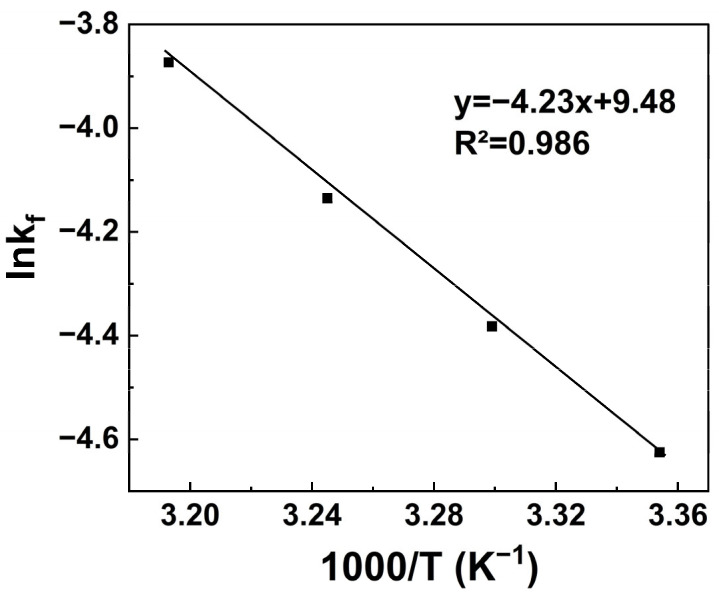
Arrhenius plot for the apparent external liquid film mass transfer coefficient *k*_f_ at 8.0% PA and 5.0% NaOH.

The effect of temperature on k_f_ was then examined under the selected condition. As shown in Figure 10, In *k*_f_ showed a linear relationship with 1000/T, with an R^2^ value of 0.986. The fitted equation was

$$lnk_{f}=-4.23\times (1000/T)+9.48$$

where T is the absolute temperature in *K*. The apparent activation energy calculated from the slope was 35.2 kJ mol^−1^. Arrhenius analysis has also been used to describe the temperature dependence of mass transfer during the extraction of phenolic compounds from plant materials [53].

When the temperature increased from 25 °C to 40 °C, *k*_f_ increased by approximately 112%. This result shows that external liquid film transfer was sensitive to temperature under the selected condition. Moderate heating accelerated the transfer of total phenolics from the particle surface into the bulk solution and reduced the relative resistance associated with the external liquid film. The temperature experiment therefore supports the rate control analysis, but it does not change the concentration condition selected from the response surface and mass transfer results.

## 4. Conclusions

Sequential PA pretreatment and NaOH extraction produced distinct effects on total phenolic release from bamboo leaves. The 4 × 4 factorial data set showed that endpoint yield increased with both factors, while the fitted transport parameters responded differently. The value of *k*_f_ was highest around 8.0% PA, whereas *D*_eff_ increased steadily with NaOH concentration. The two fitting windows captured different portions of the release curve. The LDF window, based on the 40, 60, and 80 min data, described changes in external liquid film transfer, while the Crank window, based on the 80, 100, and 120 min data, described effective intraparticle diffusion. These windows are operational ranges for parameter estimation and do not imply that the two transport processes occur separately.

The characteristic time ratio *τ*_m_/*τ*_d_ exceeded 2 under all 16 conditions, indicating that external liquid film transfer contributed the greater relative resistance within the tested system. SEM and FTIR showed treatment-related changes in surface morphology and local chemical environment, providing structural context for the kinetic trends without assigning a single structural cause. Internal validation and two additional experiments supported reliable prediction within the studied range, while extrapolation should be interpreted cautiously. Considering endpoint yield, *k*_f_, *D*_eff_, and treatment intensity, 8.0% PA and 5.0% NaOH provided the most balanced condition, yielding 18.55 ± 0.26 mg GAE/g. This study offers a practical kinetic basis for comparing transport resistance and selecting sequential extraction conditions for bamboo leaf phenolics.

## Figures and Tables

**Table 1:** PA and NaOH concentration combinations and group assignments for the kinetic experiments.

PA Concentration (%)	3.0% NaOH	4.0% NaOH	5.0% NaOH	6.0% NaOH
7	G1	G2	G3	G4
8	G5	G6	G7	G8
9	G9	G10	G11	G12
10	G13	G14	G15	G16

**Table 2:** Kinetic models used for comparison of the complete extraction curves.

Model	Abbreviation	Equation	Fitted Parameters	Parameters
Pseudo first order model	PFO	$q_{t}=q_{e}[1-exp(-k_{1}t)]$	q_e_, k_1_	2
Pseudo second order model	PSO	$q_{t}=\frac{k_{2}q_{e}^{2}t}{1+k_{2}q_{e}t}$	q_e_, k_2_	2
Peleg model	Peleg	$q_{t}=\frac{t}{K_{1}+K_{2}t}$	K_1_, K_2_	2
Weibull model	Weibull	$q_{t}=q_{e}\{1-exp[-(kt{)}^{\beta }]\}$	q_e_, k, β	3
Elovich model	Elovich	$q_{t}=a+blnt$	a, b	2

The *q_t_* is the polyphenol yield at extraction time *t*, and *q*_e_ is the fitted equilibrium yield. *k*_1_, *k*_2_, *k*, *K*_1_, *K*_2_, *a*, *b* and *β* are fitted model parameters.

**Table 3:** Endpoint total phenolic yield and mass transfer parameters under sequential PA pretreatment and NaOH extraction.

PA (%)	NaOH (%)	*q*_120_ (mg GAE/g)	*k*_f_ (min^−1^)	*D*_eff_ (×10^−12^ m^2^/s)	*τ*_m_/*τ*_d_
7	3	16.35 ± 0.19	0.02	3.00	3.52
7	4	17.40 ± 0.20	0.02	3.30	3.68
7	5	17.80 ± 0.29	0.02	3.70	3.93
7	6	18.15 ± 0.28	0.02	4.00	4.46
8	3	17.05 ± 0.23	0.03	3.20	2.85
8	4	17.95 ± 0.20	0.03	3.70	2.95
8	5	18.55 ± 0.26	0.03	4.20	3.02
8	6	19.05 ± 0.25	0.03	4.50	3.46
9	3	17.45 ± 0.23	0.02	3.40	3.29
9	4	18.30 ± 0.28	0.03	3.90	3.48
9	5	18.85 ± 0.36	0.03	4.40	3.63
9	6	19.35 ± 0.22	0.03	4.80	4.11
10	3	17.65 ± 0.25	0.02	3.50	3.90
10	4	18.55 ± 0.20	0.02	4.10	4.15
10	5	19.15 ± 0.36	0.02	4.70	4.55
10	6	19.65 ± 0.24	0.02	5.20	5.27

**Table 4:** Summary of model fitting and statistical analysis for sequential PA pretreatment and NaOH extraction.

Category	Response/Model	Metric	Result
Endpoint extraction	*q*_120_ endpoint yield	Two−way ANOVA: PA effect	*p* < 0.001
Endpoint extraction	*q*_120_ endpoint yield	Two−way ANOVA: NaOH effect	*p* < 0.001
Endpoint extraction	*q*_120_ endpoint yield	Two−way ANOVA: PA and NaOH interaction	*p* = 0.028
LDF fitting	LDF model	R^2^ range	0.971–0.993
LDF fitting	LDF model	RMSE range	0.008–0.026
LDF parameter	*k*_f_	Two−way ANOVA: PA effect	*p* < 0.001
LDF parameter	*k*_f_	Two−way ANOVA: NaOH effect	*p* = 0.002
Crank model fitting	Crank spherical diffusion model	R^2^ range	0.958–0.991
Crank model fitting	Crank spherical diffusion model	|Δa| range	0.006–0.028
Crank model parameter	*D*_eff_	Two-way ANOVA: NaOH effect	*p* < 0.001
Crank model parameter	*D*_eff_	Two-way ANOVA: PA effect	*p* = 0.0005

**Table 5:** Validation results for the two additional PA and NaOH conditions.

PA (%)	NaOH (%)	RMSE	MAE	Predicted *q*_120_	Experimental Mean *q*_120_	Relative Error of *q*_120_ (%)
7	4.5	0.04	0.032	17.6	17.57	0.15
11	7	0.171	0.142	20.2	19.94	1.27

## Data Availability

The original contributions presented in the study are included in the article and Supplementary Materials; further inquiries can be directed to the corresponding author.

## Supplementary Materials

The following supporting information can be downloaded at https://www.mdpi.com/article/10.3390/foods15152655/s1, Table S1. Total phenolic yields at different extraction times under the 16 PA and NaOH concentration combinations; Table S2. Overall fitting performance of alternative kinetic models for the complete extraction curves; Table S3. Equilibrium yield estimation and fitting statistics for the LDF and Crank spherical diffusion models; Table S4. Two-way ANOVA results for endpoint yield, *k*_f_, and *D*_eff_; Table S5. Predictive validation statistics for the 16 PA and NaOH concentration combinations.
