# Supplementary material for: Sequential Pyruvic Acid Pretreatment and Alkaline Extraction of Bamboo Leaf Phenolics: Two-Stage Mass Transfer Kinetics
Source: Foods. 2026 Jul 28;15(15):2655. doi: 10.3390/foods15152655 (PMC13463997; doi:10.3390/foods15152655)
Supplement: Supplementary file 1 [file foods-15-02655-s001.zip › foods-4442332-supplementary.pdf]

Supporting information

Sequential Pyruvic Acid Pretreatment and Alkaline Extraction of Bamboo Leaf Phenolics: Two-Stage Mass Transfer Kinetics

Yongkang Mo [a], Xiaoying Liu [a], Jingpeng Zhou [b], Jianquan Ren [a], Baojie Liu [a], Chengrong Qin [a], Chen Liang [a], Caoxing Huang [c], Shuangquan Yao [a*]*

[a] Guangxi Key Laboratory of Clean Pulp & Papermaking and Pollution Control, School of Light Industrial and Food Engineering, Guangxi University, Nanning, 530004, PR China

[b] Shandong Huatai Paper Co., Ltd. Dongying & Shandong Key Laboratory of Biobased Material and Green Pulp Papermaking, Dongying 257335, China

[c] Jiangsu Co-Innovation Center of Efficient Processing and Utilization of Forest Resources, Nanjing Forestry University, Nanjing 210037, PR China

[*]Corresponding author: yaoshuangquan@gxu.edu.cn

List of Supporting information

Table S1. Total phenolic yields at different extraction times under the 16 PA and NaOH concentration combinations.

Table S2. Overall fitting performance of alternative kinetic models for the complete extraction curves.

Table S3. Equilibrium yield estimation and fitting statistics for the LDF and Crank spherical diffusion models.

Table S4. Two-way ANOVA results for endpoint yield, k_f, and D_{eff}.

Table S5. Predictive validation statistics for the 16 PA and NaOH concentration combinations.

Table S1. Total phenolic yields at different extraction times under the 16 PA and NaOH concentration combinations.

PA (%)	NaOH (%)	Time(min)	Yield (mg GAE/g)
7	3	40	13.40±0.13
7	3	60	14.70±0.13
7	3	80	15.55±0.18
7	3	100	16.40±0.28
7	3	120	16.35±0.19
7	4	40	14.05±0.14
7	4	60	15.30±0.20
7	4	80	16.35±0.24
7	4	100	17.25±0.28
7	4	120	17.40±0.20
7	5	40	14.45±0.16
7	5	60	15.60±0.22
7	5	80	16.95±0.21
7	5	100	17.85±0.26
7	5	120	17.80±0.29
7	6	40	14.75±0.15
7	6	60	15.95±0.17
7	6	80	17.30±0.30
7	6	100	18.20±0.25
7	6	120	18.15±0.28
8	3	40	13.95±0.14
8	3	60	15.20±0.23
8	3	80	16.45±0.27
8	3	100	17.10±0.19
8	3	120	17.05±0.23
8	4	40	14.60±0.11
8	4	60	15.75±0.19
8	4	80	17.15±0.17
8	4	100	17.90±0.32
8	4	120	17.95±0.20
8	5	40	15.10±0.15
8	5	60	16.30±0.17

8	5	80	17.80±0.27
8	5	100	18.50±0.35
8	5	120	18.55±0.26
8	6	40	15.50±0.19
8	6	60	16.70±0.26
8	6	80	18.25±0.22
8	6	100	19.00±0.36
8	6	120	19.05±0.25
9	3	40	14.25±0.10
9	3	60	15.45±0.18
9	3	80	16.80±0.23
9	3	100	17.40±0.22
9	3	120	17.45±0.23
9	4	40	14.90±0.17
9	4	60	16.10±0.13
9	4	80	17.55±0.17
9	4	100	18.25±0.28
9	4	120	18.30±0.28
9	5	40	15.35±0.16
9	5	60	16.50±0.20
9	5	80	18.10±0.28
9	5	100	18.80±0.30
9	5	120	18.85±0.36
9	6	40	15.80±0.16
9	6	60	17.05±0.24
9	6	80	18.65±0.18
9	6	100	19.30±0.36
9	6	120	19.35±0.22
10	3	40	14.55±0.11
10	3	60	15.80±0.16
10	3	80	17.10±0.23
10	3	100	17.70±0.33
10	3	120	17.65±0.25
10	4	40	15.20±0.16
10	4	60	16.35±0.21
10	4	80	17.85±0.27
10	4	100	18.50±0.33
10	4	120	18.55±0.20
10	5	40	15.65±0.11
10	5	60	16.70±0.16
10	5	80	18.40±0.27
10	5	100	19.10±0.25
10	5	120	19.15±0.36
10	6	40	16.05±0.20
10	6	60	17.25±0.20

10	6	80	18.90±0.31
10	6	100	19.60±0.34
10	6	120	19.65±0.24

Table S2. Overall fitting performance of alternative kinetic models for the complete extraction curves.

Model	Abbreviation	Mean R^2	Mean RMSE	Mean MAE	Mean AICc	Mean BIC	AICc best count
Pseudo first order model	PFO	0.973	0.216	0.199	-5.452	-12.234	6
Pseudo second order model	PSO	0.973	0.216	0.199	-5.452	-12.234	9
Peleg model	Peleg	0.966	0.242	0.214	-4.319	-11.101	1
Weibull model	Weibell	0.945	0.308	0.247	-1.887	-8.668	0
Elovich model	Elovich	0.975	0.207	0.192	14.064	-11.108	0

Table S3. Equilibrium yield estimation and fitting statistics for the LDF and Crank spherical diffusion models.

PA (%)	NaOH (%)	q∞ (mg GAE/g)	q∞ SD	R^2	RMSE	R^2 LDF	RMSE LDF	R^2 Crank	RMSE Crank
7	3	16.90	0.19	0.988	0.090	0.986	0.025	0.988	0.024
7	4	17.54	0.21	0.991	0.096	0.988	0.027	0.978	0.018
7	5	18.18	0.18	0.987	0.102	0.972	0.029	0.984	0.022
7	6	18.82	0.22	0.990	0.108	0.974	0.021	0.990	0.026
8	3	17.62	0.17	0.991	0.096	0.988	0.027	0.988	0.024
8	4	18.36	0.20	0.987	0.102	0.972	0.029	0.978	0.018
8	5	19.20	0.23	0.990	0.108	0.974	0.021	0.984	0.022
8	6	19.74	0.25	0.986	0.114	0.976	0.023	0.990	0.026
9	3	17.88	0.22	0.987	0.102	0.972	0.029	0.988	0.024
9	4	18.67	0.23	0.990	0.108	0.974	0.021	0.978	0.018
9	5	19.46	0.24	0.986	0.114	0.976	0.023	0.984	0.022
9	6	19.90	0.26	0.989	0.090	0.978	0.025	0.990	0.026
10	3	18.05	0.23	0.990	0.108	0.974	0.021	0.988	0.024
10	4	18.84	0.22	0.986	0.114	0.976	0.023	0.978	0.018
10	5	19.68	0.27	0.989	0.090	0.978	0.025	0.984	0.022
10	6	20.17	0.28	0.992	0.096	0.980	0.027	0.990	0.026

Table S4. Two-way ANOVA results for endpoint yield, k_f, and D_{eff}.

Response variable	Source	df	F value	p value	Interpretation
Endpoint yield at 120 min	PA	3	62.4	<0.001	PA significantly affects endpoint yield
Endpoint yield at 120 min	NaOH	3	188.6	<0.001	NaOH is the dominant factor for final yield
Endpoint yield at 120 min	PA × NaOH	9	2.85	0.018	Interaction supports non-additive process response
k_f	PA	3	246.5	<0.001	PA mainly modulates early-stage film transfer
k_f	NaOH	3	18.2	<0.001	NaOH has a secondary effect on k_f
k_f	PA × NaOH	9	5.4	<0.001	PA effect depends partly on alkali background
D_{eff}	PA	3	31.7	<0.001	PA assists internal accessibility
D_{eff}	NaOH	3	280.2	<0.001	NaOH strongly enhances intraparticle diffusion
D_{eff}	PA × NaOH	9	2.1	0.054	Interaction is weaker for D_{eff} than for k_f

Table S5. Predictive validation statistics for the 16 PA and NaOH concentration combinations.

PA (%)	NaOH (%)	RMSE	MAE	Experimental mean q_{120}	Predicted q_{120}	Relative error of q_{120} (%)
7	3	0.178	0.147	16.35	16.62	1.62
7	4	0.157	0.142	17.4	17.21	1.07
7	5	0.168	0.142	17.8	17.9	0.54
7	6	0.257	0.201	18.15	18.53	2.11
8	3	0.109	0.106	17.05	17.12	0.42
8	4	0.156	0.138	17.95	17.9	0.26
8	5	0.167	0.149	18.55	18.55	0.03
8	6	0.221	0.199	19.05	19.01	0.23
9	3	0.148	0.095	17.45	17.58	0.76
9	4	0.151	0.109	18.3	18.37	0.36
9	5	0.222	0.148	18.85	19.04	1.02
9	6	0.171	0.159	19.35	19.54	0.99
10	3	0.182	0.157	17.65	17.76	0.64
10	4	0.166	0.151	18.55	18.57	0.1
10	5	0.22	0.172	19.15	19.27	0.61
10	6	0.195	0.19	19.65	19.77	0.61
